# You might as well smoke; the misleading and harmful public message about smokeless tobacco

**DOI:** 10.1186/1471-2458-5-31

**Published:** 2005-04-05

**Authors:** Carl V Phillips, Constance Wang, Brian Guenzel

**Affiliations:** 1 University of Texas Medical School, Center for Clinical Research and Evidence Based Medicine, Houston, USA; 2 University of Texas School of Public Health, Houston, USA; 3 Center for Philosophy, Health, and Policy Sciences, Inc., 10923 Atwell Dr, Houston, TX 77096, USA

## Abstract

**Background:**

Compared to smoking cigarettes, use of Western smokeless tobacco (ST) products is associated with a very small risk of life-threatening disease (with estimates in the range of a few percent of the risk from smoking, or even less). This means that smokers can realize substantial health benefits by switching to ST, an obvious substitute. But consumers and policy makers have little chance of learning that ST is much less dangerous than smoking because popular information provided by experts and advocates overstates the health risks from ST relative to cigarettes.

**Methods:**

To examine the extent of this overstatement in one medium, we conducted a systematic review of websites containing information about ST and health risks. We examined the content of 316 relevant websites identified by a Google search.

**Results:**

We found that when any substantive information about the risk from ST is given, the risk is almost universally conflated with the risk from cigarettes. Accurate comparative risk information was quite rare, provided by only a handful of websites, all appearing low in our search results (i.e., of low popularity and thus unlikely to be found by someone searching for information). About 1/3 of the websites, including various authoritative entities, explicitly claimed that ST is as bad as or worse than cigarettes. Most of the other sites made statements that imply the risks are comparable.

**Conclusion:**

Through these websites, and presumably other information provided by the same government, advocacy, and educational organizations, ST users are told, in effect, that they might as well switch to smoking if they like it a bit more. Smokers and policy makers are told there is no potential for harm reduction. These messages are clearly false and likely harmful, representing violations of ethical standards.

## Background

The negative health consequences of smoking cigarettes are well known. What is less well known is that not all tobacco products create similar levels of risk. In particular, use of Western smokeless tobacco (ST) is substantially less harmful than smoking cigarettes. This should not be surprising, given that ST use does not expose the body to the harmful combustion products and assault on the lungs that result from smoking. But even many health experts do not realize there is a major difference, perhaps because of repeated messages about "tobacco" (usually referring just to cigarettes), which imply that all products made from this plant have the same health implications.

ST is usually only linked to one life-threatening disease, oral cancer (OC), and even that association may not apply to the types of products that increasingly dominate ST use in the West [1]. Claims of OC risk are largely based on a single study [2] and are contradicted by a substantial portion of the evidence about modern moist snuff [3-6]. Claims are sometimes also made about links to cardiovascular disease and pancreatic cancer, though the evidence supporting these claims is even thinner and more equivocal. The lack of clear evidence of a strong association with any diseases is not due to lack of research; there have been extensive attempts to find health risks from ST, including in Swedish populations where prevalence of use is high. While it is impossible to ever rule out small associations between an exposure and a disease, there is ample evidence to rule out, with a very high degree of confidence, the possibility that the combined risk of life threatening diseases due to ST use is anything close to that from smoking.

Even if we were to believe the commonly cited estimates for the risk of OC from ST, that risk is still lower than the estimated risk due to smoking for OC alone (a very small fraction of the total risk from smoking). If we further allow for the possibility that ST creates small, yet-undetected risks for some other diseases, the risks from the many diseases caused by smoking still clearly dwarf possible risk from ST. The most frequently repeated estimate conservatively puts the risk of premature mortality from ST use at 2% of that from cigarettes [7,8]. The Royal College of Physicians recently stated that the risk from ST might be as low as 1/1000 that from cigarettes [9]. The Royal College and another recent high-profile report suggest an upper bound estimate of 1/10 the risk [10], but the available epidemiology suggests that the true value is extremely unlikely to be this large. Whatever the exact magnitude, the conclusion must be that cigarettes are considerably more harmful than ST.

This comparison is more than a matter of curiosity or perspective; the products are obvious substitutes. Using products that provide nicotine (which is, in itself, fairly benign) need not be very harmful – it is smoking that is very harmful. Among the several things a smoker can do to eliminate most of the risk from his nicotine use (e.g., quitting nicotine entirely or using pharmaceutical nicotine products), switching to ST is unique in allowing continued consumption of nicotine using a product for which there is a history of consumer demand. For some smokers, this switch – a "harm reduction" strategy – offers the best chance of changing their behavior to eliminate the huge risk from smoking. Calls for such a strategy are increasing in popularity among advocates and in the media.

However, if smokers are unaware of the difference in risk between the two products they are obviously unlikely to switch from cigarettes to ST to reduce their risk. Moreover, if current ST users are unaware of the difference, they will believe that they might as well smoke, possibly resulting in a terribly unhealthy change of products. Casual empiricism and what data exists suggest that consumers are largely unaware that there is a large (or any) difference in risk, and popular information reinforces that lack of understanding. Inaccurate statements by experts, government organizations, and advocates that appear in the press and educational materials frequently reinforce the mistaken belief that risks are similar. To expand on casual observations about such misinformation and to systematically assess its prevalence in one forum – web pages offering health advice about ST – we conducted the systematic review presented in this paper.

### Unethical messages

A preeminent tenet of modern health and medical ethics is the right of individuals to make fully-informed autonomous decisions, and the accompanying obligation of health experts, clinicians, and policy makers to provide the information and permit the autonomy. Systematic provision of inaccurate comparative risk information violates these principles.

The provision of such misinformation can be attributed to ignorance of the science, deliberate misrepresentation under the belief that it is for people's own good, or deliberate misrepresentation for other reasons. Each of these is an ethical violation. Intentionally misleading people about health information, even for their own good, is considered ethically unacceptable in all but the most extreme emergent cases, and in the present case there are compelling arguments that this misleading information does more harm than good. But even when ignorance is the explanation, it must be considered unethical. When an individual or organization actively endeavors to provide authoritative information and portray it as accurate, particularly when such an entity is considered a respected authority on health science, ignorance of well-established scientific truth is unethical negligence.

Kozlowski and O'Conner recently challenged the U.S. Centers for Disease Control and Prevention (CDC) and the U.S. Substance Abuse and Mental Health Services Administration (SAMHSA) over the content of their websites, which included clearly false claims that ST poses a similar health risk to cigarettes [11]. Kozlowski and O'Conner reported that following their protests, CDC changed their website (SAMHSA did not), though the change was merely from out-and-out falsehood ("Is smokeless tobacco safer than cigarettes? NO WAY!") to a literally true statement that still misleads readers, a tactic discussed below. Our review lets us assess how pervasive such false and misleading comparisons are.

## Methods

To examine the extent of systematic overstatement of the risks from ST in one medium, we conducted a systematic review of popular sources of information, looking at websites that implicitly purport to deliver a public service message about ST and health risks. (We have previously presented preliminary results of this research along with more extensive background [12,13].)

With an increasing portion of those seeking health information turning to the web,[14,15] information found there provides a good measure of what people might learn. While websites do not contain all popularly available information, many people searching for information on this topic would start with a web search and most organizations that have a stated position on the topic, particularly those actively trying to influence popular opinion through other media, have a web page that reflects their claims. Thus, the information in web pages is likely to be representative of all information reaching the average consumer.

We performed a Google search for [tobacco AND cancer AND (smokeless OR snuff OR dip OR spit OR chew OR chewing)], the latter disjunction covering most of the synonyms for "smokeless". We conducted the search on 3 May 2003 and stored the results offline so they would not change when re-accessed. The search reported 763 results (after Google's algorithms eliminated many, but not all, multiple similar hits), which we used as our dataset. While the nature of the Google search makes it impossible to describe the exact sampling properties that yielded this population, and thus we would hesitate to do any formal statistical analysis, it is safe to conclude that it contains all of the most popular websites that address the subject, as well as a large portion of the less popular ones. This is sufficient for the present analysis.

We were interested in public service sites (as implicitly self-defined, without an attempt on our part to judge what constitutes a genuine public service) or health advice/information sites which state an entity's opinion about the health risks from ST. Our protocol eliminated: sites that were selling tobacco products or methods for quitting tobacco (with one exception discussed below); news of the day; search engine, web maps, and other sites that just provide links; sites from South Asia (because the products used there contain major ingredients other than tobacco and the epidemiologic research suggests they create greater oral health problems than Western moist snuff); and scientific literature (scholarly papers, journals, and conference abstracts). Eliminating these and the double counting from organizations that were duplicated in the Google results yielded 316 web presences in our population. The search terms limited the results to English language sites. The vast majority were U.S. entities, with a handful from the U.K., Canada, and other countries.

For each included website, we searched the entire website (not just the page hits from the Google search) for statements about the health effects of ST and collected the results. We ignored information that was clearly not the position of the sponsoring organizations (e.g., quotations of material they were disagreeing with; postings on message boards). We initially reviewed the websites between 4 May 2003 and 11 June 2003 and printed out relevant pages. We audited our results and expanded the collected data during the period 25 September 2003 through 8 December 2003. To maximize consistency, multiple researchers viewed each website, and ambiguous codings were discussed by the group.

The ordering of the websites in our list is important because those that are earlier are more popular (specifically, are more often linked to from other sites) and are much more likely to be found and accessed by someone doing a search. In the results presented below, the hit number is a website's ranking within in our list of 763, with lower numbers being higher ranked (earlier in the list). For websites that generated multiple hits, we present the highest ranked hit unless otherwise noted.

## Results and Discussion

The risk from ST is widely conflated with the risk from cigarettes on websites that provide health advice and information. Almost every website had statements that played up the health risks from ST without caveat, making it difficult for consumers to recognize the huge contrast with cigarettes. The quantitative claims of health risks from ST were very often beyond a worst-case-scenario interpretation of the scientific literature. A large portion of websites directly stated or implied that the risks from ST and cigarettes are similar.

As noted above, the most salient feature of the comparative risks of smoking and ST is how different they are, a message that is buried deeply in the websites that inform the public on this topic.

### Very little accurate comparative risk information

Very few websites provided accurate information. Two organizations, ASH (Action on Smoking and Health) in the U.K. and the American Council on Science and Health (ACSH) in the U.S. were the most prominent sources of accurate comparative risk information. Their highest appearances in our search were hits 96 and 93, respectively, meaning they would likely not be found by someone seeking information on ST, since most people seldom read beyond the first few tens of hits [16,17]. Moreover, it was not until hit 491 that ASH's major harm reduction statement [10], probably the most prominent current call to consider harm reduction for cigarettes, appears. ACSH's harm reduction message appears at hit 120 [16]. The earlier hits were actually anti-harm-reduction statements, apparently presented by those organizations to acknowledge other positions (these ranked higher that the organizations' actual stated positions because more other websites linked to those pages).

Brad Rodu, a professor of pathology and dentistry at the University of Alabama at Birmingham (UAB), is the longtime leading advocate of the use of ST as a harm reduction strategy for smoking. His pages at the UAB website provide comprehensive information on the topic, but this was only hit 625 on our list [18]. A commercial site (included because of its extensive health message) for a quit-smoking product, hit 408, is a mirror of an old version of Rodu's UAB pages, posted under Rodu's name with his permission [19]. Despite his numerous writings on the topic, the only earlier entries on our list that would lead to Rodu's work were hit 276, one of his op-eds in the news archives from an anti-tobacco organization, and the aforementioned ACSH hit 120 [16].

Only three other sites mentioned that ST use is not as bad as cigarettes, and they offered little more than mentions [20-22]. Astonishingly, we were unable to find any other statements about the much lower risk of ST compared to smoking. No high-ranking sites provided the information tobacco users would need to make choices based on which product is safer. Notably, no site from the most prolific source of information, the U.S. government, provided such information (excluding a few scholarly or technical papers that can be downloaded from the sites but are not presented as the government's message to consumers). Indeed, U.S. government agencies consistently provided misleading information, as did popular medical advice sites and the best-known advocacy groups.

### Misleading comparative risk information

The most prevalent messages were those that would tend to convince readers that the health risk from ST is comparable to that from smoking.

We identified 237 of the remaining 309 websites in our population as discussing the risks of smoking and ST in proximity to each other. Most of the other 72 sites either contained very little substance (often just a passing mention that ST poses health risks), appeared very low in our results, or both, so these numbers tends to understate how common the juxtaposition of health claims about cigarettes and ST is. Any juxtaposition of health claims about the two products that does not make clear the very different absolute risk, even if it makes no explicit comparison, implies to readers that the risks are comparable. Most websites did more than merely juxtapose; they made specific statements that reinforced this implication.

### Explicit claims of equal risk

We identified 108 websites that claimed that the risks from ST are as bad as or worse than those from smoking. Most often this took the form of an explicit statement that ST is not safer than smoking. It is worth noting that this is equivalent to saying that you are better off, or at least no worse off, deciding to smoke rather than use ST.

#### Examples include various authoritative entities

• American Cancer Society: "Some people believe that using smokeless tobacco is safer than smoking. This is not true." [23]

• World Health Organization: "There is also a prevalent myth that it is less dangerous than smoking. The reality is that smokeless tobacco is just as addictive and fatal as cigarettes." [24]

• U.S. Department of Health and Human Services (the statement noted by Kozlowski and O'Conner): "Q. Isn't smokeless tobacco safer to use than cigarettes? A. No." [25]

### Implicit claims that ST is worse than cigarettes

Of the 108 websites making claims that ST is as bad or worse than cigarettes, 26 suggested that ST is worse than smoking by likening the risks and then identifying differences that exclusively favor smoking.

A typical example appears in the second highest-ranking website from our search, the Academy of General Dentistry: "Isn't it safer than smoking? Absolutely not. Some wrongly believe that spit tobacco is safer than smoking cigarettes. But spit tobacco is more addictive because it contains higher levels of addictive nicotine than cigarettes and can be harder to quit than cigarettes. One can of snuff delivers as much nicotine as 60 cigarettes." Though there is no explicit claim that ST is worse – the explicit claim is simply that it is no better – the comparisons that follow imply that it is better to smoke than to use ST.

### Implicit claims of equal risk

Of the websites not making explicit claims that ST is as bad as or worse than cigarettes, 100 made statements that directly imply that risks from ST are comparable to those of smoking, while another 29 simply juxtaposed the two risks without suggesting there are differences. (Most of those that made explicit claims also included some of these implicit claims.)

There are various literally true statements that are apparently intended to dissuade readers from the (accurate) belief that ST is safer than smoking. Some might argue that such statements do not violate ethical rules that prohibit lying. On the other hand (as has been widely discussed regarding recent U.S. government policy in other arenas), clearly misleading statements that are carefully crafted to be literally true are arguably worse than literally false statements. They suggest that the authors know the truth and believe that it is sufficiently clear that they should maintain a plausible claim they are not contradicting it, but are nevertheless trying to get people to believe a falsehood.

The most popular types of literally-true-but-misleading information were comparisons with smoking that characterize ST as "not a safe substitute to smoking cigarettes" or "not harmless," or statements that "there is no safe tobacco." (The former of these is quoted from the 1986 U.S. Surgeon General's report [27] or the similar warning on 1/3 of the units of ST products sold in the U.S.) We identified 62 websites (among those not making an explicit claim of equivalent risk) that made one or more such claims. Since basically nothing is perfectly safe, these statements are literally true, but the comparison implies more than the literal interpretation, "it would not eliminate every last bit of risk to switch from cigarettes to ST." Saying "ST is not a safe alternative" without any hint of the fact that it is *immensely safer *implies that there is no benefit in switching from smoking to ST or, equivalently, no increased risk in switching from ST to smoking.

We identified 55 websites where ST and smoking risks were combined in lists of health effects or attributable risk (this excludes those that make explicit claims of equivalent risks, but overlaps with the 62 in the previous paragraph), either by conjunction or by using the word "tobacco" in contexts where it refers to both products. A popular U.S. health advice site, Virtual Hospital, states, "Both cigarettes and smokeless tobacco are harmful to your child's health," followed immediately by detailing the known health effects of smoking [28]. The U.S. National Library of Medicine's consumer advice site, MedlinePlus, under the heading "Tobacco use, smoking and smokeless tobacco," states, "Tobacco and its various components increase the risk of cancer (especially in the lung, mouth, larynx, esophagus, bladder, kidney, pancreas, and cervix), heart attacks and strokes, and chronic lung disease" [29]. Absent a statement of specific or comparative risks, this tends to imply that the components of the conjunction contribute similarly to all the claimed outcomes. These conjunctions are particularly common in the later Google hits, which only briefly mention ST, often in a broad discussion of behavioral risk factors, suggesting that most brief presentations of the health effects of ST conflate the exposure with smoking.

The U.S. National Cancer Institute (NCI) had the largest number of search hits (all of the first 4 and 16 others). We found no literally false claims in the NCI websites. However, they did make many literally true misleading claims, including saying "not a safe alternative" [30-32], and lumping together attributable risk from ST for oral cancer with the (many times greater) attributable risk from cigarettes [32,33]. A particularly misleading conjunction is, "Smoking tobacco, using smokeless tobacco, and being regularly exposed to environmental tobacco smoke are responsible for one-third of all cancer deaths in the United States each year" [34]. Even the worst-case scenario for claims about the risk from ST would make it responsible for in the order of 1/1000 of this attributable risk.

### Relative popularity

The imbalance of good and bad information is somewhat worse if we focus on the hits from earlier in the list (i.e., the ones more likely to be found and accessed). Looking at the first 90 hits, chosen because (at the default ten hits displayed per page) those would be appear before a searcher saw a link to ASH, ACSH, or any accurate comparative risk information, yields 44 websites in our population. Those include 13 that claim ST is as bad or worse than cigarettes and 19 others that use one of the rhetorical devices to imply the risks are similar.

## Conclusion

Even though we were aware that the available popular information was skewed before we undertook the systematic review, we were astonished to find the near ubiquity of misinformation and how unlikely consumers are to find accurate comparative risk information. Websites provide a substantial and growing portion of the knowledge consumers get about health issues, including the health implications of tobacco use. We expect that the mix of information about ST we found is similar to that provided in pamphlets, public service messages, and other popular media since the organizations represented in our websites are the same ones that provide that information. A recent study reporting some information about pamphlets tends to confirm this hypothesis [35], as does our monitoring of popular press stories (unpublished). Thus, the pattern of misinformation we found very likely represents a comparable pattern of misinformation reaching people from all popular sources.

The negative health implications of preventing people from realizing that ST is relatively safe should not be underestimated. ST users are told, in effect, that they might as well switch to smoking if they find they like it a bit more. The larger population of smokers is told that they cannot switch to a safer form of tobacco, a message that is often characterized as "quit or die." It is extremely difficult for anyone to deliver a harm reduction message in the face of the widespread misperception that is fueled by the misinformation. At this point, we can only speculate about how many smokers would take advantage of this opportunity to reduce their risk by two orders of magnitude or more.

Health advocates, particularly those in public service, have an affirmative ethical duty to tell the truth. It is difficult to justify keeping the truth from people, even when knowing it might be harmful; it is clearly unjustified when it would be beneficial.

## Abbreviations

ST = smokeless tobacco

OC = oral cancer

CDC = U.S. Centers for Disease Control and Prevention

ASH = Action on Smoking and Health

ACSH = American Council on Science and Health

UAB = University of Alabama at Birmingham

NCI = U.S. National Cancer Institute

## Competing interests

Phillips is the director of and Guenzel was (at the time of writing) employed by the non-profit research institute, Center for Philosophy, Health, and Policy Sciences, Inc. CPHPS is the recipient of an unrestricted gift from U.S. Smokeless Tobacco Company for support of research of Dr. Carl V. Phillips. This research was investigator-initiated. USSTC did not influence the content or see the study results before they were publicly released. Phillips and Wang have received consulting fees from USSTC related to litigation. Phillips is the recipient of an unrestricted research grant from USSTC at the University of Alberta where he will soon be employed.

## Authors' contributions

CVP designed the study and analysis, participated in data collection and cleaning, and wrote the manuscript (based partially on earlier poster versions). CW assisted in the design of the study, participated in data collection and analysis, and oversaw the data cleaning and auditing. BG was the primary data collector, contributed to study design, participated in data cleaning and analysis, and coauthored the earlier poster versions of the manuscript. All authors read and approved the final manuscript.

## Pre-publication history

The pre-publication history for this paper can be accessed here:


